# “Are you there?”: Teaching presence and interaction in large online literature classes

**DOI:** 10.1186/s40862-022-00180-3

**Published:** 2022-12-16

**Authors:** Fang Li

**Affiliations:** College of International Studies, Southwest University, Chongqing, China

**Keywords:** Teaching presence, Social presence, Cognitive presence, Online interaction, Literature classes

## Abstract

Increasing interaction in large online classes is a challenge that many teachers are facing in the post-pandemic era. This study, rooted in Garrison et al.’s CoI (Community of Inquiry) framework, employs both quantitative and qualitative methods to explore what a teacher can do in large online literature classes to promote interaction by way of enhancing teaching presence. The correlation and regression analysis of the questionnaire survey indicates that the teacher’s strategies lead to high levels of teaching, social and cognitive presence, and in turn facilitate students’ online interaction, resulting in their strong sense of satisfaction. Besides, it suggests teaching presence has stronger relationship with cognitive presence than social presence. In addition, social and cognitive presences are strong predictors for learning outcomes which account for 68% of the explained variance in this study. Students’ online interaction in the form of postings show that they are more cognitively engaged rather than socially involved, which implies that students are more focused on the construction of knowledge rather than try to be connected in the community. The limited peer interaction in spite of students’ acknowledgement that peer interaction plays a unique role in pushing them towards better understanding of the texts poses the teacher another challenge for enhancing social presence.

## Introduction

Since 2020, the COVID-19 pandemic has gradually brought traditional face-to-face teaching to various online platforms. Though these platforms have become very convenient media connecting teachers and students wherever they are, still, teachers are facing huge challenges to involve students in E-learning with the seemingly cold screen discouraging them from having expressive warm face-to-face interaction. As Sjølie et al.(2022) report in their research that “the digital medium acted as a filter in the communication, reducing students’ ability to see other team members’ body language and non-verbal social cues”, causing it difficult to “interpret verbal messages and to read the entire emotional register”. E-learning in super-sized classes particularly causes challenges for interaction and maintaining presence (Nagel & Kotze, 2010). Besides, part-time students in this case especially face problems caused by combining study with other commitments like jobs and families (Deris, 2012). Teaching literature to large class online students can be extremely daunting if students are neither reading nor thinking hiding behind the screen leaving the teacher to be the sole speaker for hours without any idea about whether students are there at all so that the teacher is likely to end up being exhausted with little sense of achievement. Therefore, to survive as a literature teacher of distant education, it’s essential to make sure the students are there without asking the dumb question “Are you there?”—the sense of students’ presence will serve to empower and energize the teacher, enabling them to move on with the class. “Being there” in the virtual environment conveys the message that the teacher is speaking to real people and senses the connection with students, which may in turn be mirrored by students’ interaction with the teacher or other participants. But what can the literature teacher do to make sure the students are there for the course and fully involved in class activities so that satisfying learning outcomes may be achieved? This study aims to answer the question by focusing on teaching presence to bridge the gap between the teacher and the participants caused by the screen and promote students’ involvement in E-learning. The concept of teaching presence is adopted from Canadian scholar Garrison’s CoI (Community of Inquiry) framework, which involves the interplay of three presences as shown in the following figure and will be applied to this study:
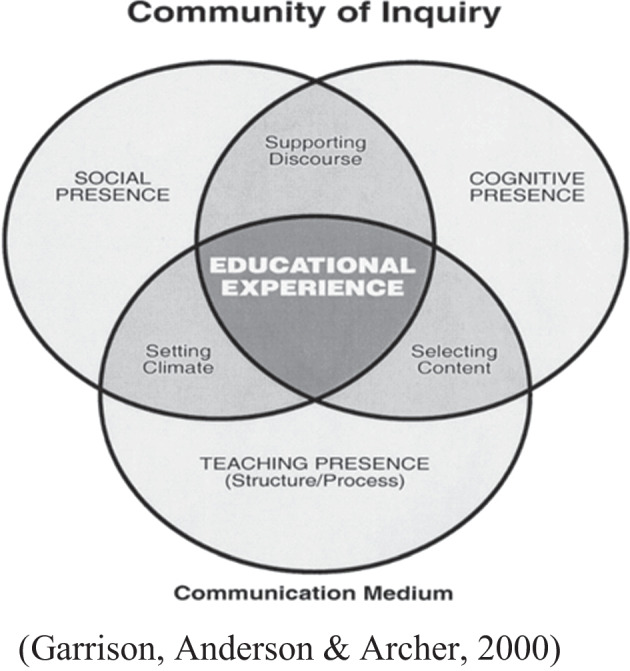


As D.R. Garrison points out in the third edition of his most influential work *E-Learning in the 21st Century*, “At the educational core is an awareness that students need to be engaged in sustainable learning communities that support reflective discourse and deep approaches to learning” and “The goal of E-learning was to explore the creation of communities of learners who could remain connected independent of time and location through the use of information and communication technologies” (2017), it’s crucial for teachers to learn how to create learning communities where students feel comfortable to be present and engage in productive thinking, which is exactly what this study means to explore. Garrison et al. define teaching presence as “a means to an end, to support and enhance social and cognitive presence for the purpose of realizing educational outcomes” (2000), therefore, it is “core to establishing and maintaining social and cognitive presence” (2010) and has the “central role” to “establishing and sustaining an online learning environment and realizing intended learning outcomes” (2010).Therefore, to understand teaching presence, one has to understand social and cognitive presence as well. Social presence is regarded as “the ability of the participants in the Community of Inquiry to project their personal characteristics into the community, thereby presenting themselves to the other participants as ‘real people’” (Garrison, 2000), which emphasizes participants’ sense of connection with the others through the role of community. Garrison et al. believe that teaching presence is essential in establishing a sense of social presence by engendering an atmosphere of trust, open communication and group cohesion (2010). Different from social presence, cognitive presence refers to “the extent to which the participants in any particular configuration of a community of inquiry are able to construct meaning through sustained communication” (2000). CoI framework emphasizes conversations among participants and leads to epistemic engagement and productive discourse (Shea & Bidjerano, 2009). Social presence and cognitive presence are related to students’ affective and cognitive involvement, which can be regarded as predictors for student involvement in class. As Song (2022) emphasizes, “effective online teaching requires building social relationships and establishing emotional security to promote dialogue and create a collaborative learning community”, this study hopes to explore the strategies that may be employed by the teacher to enhance his/her teaching presence and promote social and cognitive presence in large online literature classes, eventually bringing student involvement into reality.

## Literature review

### Teaching presence and online interaction

Following the CoI framework established by Garrsion et al. (2000), studies on teaching presence in E-learning have documented the vital role of teaching presence in facilitating students’ participation and proved that interaction is the essence of a community of inquiry experience (Shea, et al., 2005, 2006; An et al., 2009; Gorsky et al., 2010; Ke, 2010; Morueta et al., 2016; Garrison, 2017; Turk, 2022). Some scholars have also tried to explore ways to increase teaching presence so that student involvement may be promoted. For example, Dringus, et al. (2010) find that mini audio presentations can be effective ways to enhance teaching presence and encourage student participation; Deris et al. (2012), focusing on teaching presence in an online course for part-time undergraduates, discover that “careful planning of a course, and effective discourse facilitation and direct instruction, with emphasis on teachers’ personal presence, are fundamental in making presence felt in the online classroom”; Nagel and Kotze (2010), employing the CoI framework, emphasize that innovative use of Information and Communications Technology(ICT), such as electronic peer review and extensive feedback can help provide excellent teaching presence and contribute to student engagement in large online classes. These studies have both provided illuminating insights into teachers’ role in promoting online interaction and left plenty of space for further exploration regarding to different courses and subject groups.

### Social presence, cognitive presence and online interaction

Compared to the relationship between teaching presence and interaction, scholars showed more concern on the relationship between social, cognitive presence and online interaction. Social and cognitive presences are fundamental to online interaction, which may serve as a bridge between teaching presence and student engagement since one of the major roles of teaching presence lies in bringing about social and cognitive presences. In fact, social presence cannot exist without interpersonal interaction (Kehrwald, 2008), the concept of social presence itself entails how it may contribute to interaction. As social presence refers to “the degree to which a person is perceived as a ‘real person’ in mediated communication” (Gunawardena, 1995) and means “creating a climate that supports and encourages probing questions, skepticism and the contribution of explanatory ideas”(Garrison, 2017), creating social presence is equal to promoting interaction and directs to “a willingness on the part of participants to engage in communication exchanges”(Lowenthal, 2009). Short et al., who initiated social presence theory (1976), consider social presence as “a subjective quality of the medium itself” and believe that, “Media having a high degree of social presence are judged as being warm, personal, sensitive and sociable”. Short et al.’s conception of social presence changes media into something approachable in distance education, which has laid foundation for interaction. Following Short et al.’s research on social presence, many scholars started to turn their attention to social presence in distance learning and their researches always employed interaction as an important predicator of social presence (Gunawardena & Zittle, 1997; Lowenthal, 2009; Kehrwald, 2008;Weidlich & Bastiaens, 2019; Garrison et al., 2009; Lehman & Conceição, 2010; Kim, 2016; Kreijns et al., 2022, etc.). Garrison et al.’s studies on social presence imbedded in the CoI framework (2000) have been the most influential, which also regard social presence as “communication medium”, and define it as “the ability of the participants in the Community of Inquiry to project their personal characteristics into the community, thereby presenting themselves to the other participants as ‘real people’”(2000). This definition shares Short et al.’s idea of “intimacy” through the emphasis of “personal characteristics” and emphasizes participants’ sense of connection with the others through the role of community. For Garrison et al., social presence functions as “a support for cognitive presence, indirectly facilitating the process of critical thinking carried on by the community of learners” and “is a direct contributor to the success of the educational experience”(2000). Therefore, social presence prepares for cognitive presence and works together with it to help students achieve learning outcomes. Eventually, social presence contributes to the perceived satisfaction of relatedness with its affective expression, open communication, and group cohesion components (Turk et al., 2022). Tu and Mcisaac(2002), redefining social presence as “the degree of feeling, perception, and reaction to another intellectual entity in the CMC environment”, believe that “social presence is a vital element influencing online interaction” and “necessary to enhance and foster online interaction”. They consider social context, online communication and interactivity as three dimensions of social presence. Therefore, to increase social presence entails strategies to improve the three dimensions.

Although the definition of social presence has been under debate over the years (Lowenthal, 2009; Kehrwald, 2008; Kim, 2016; Kreijns et al.,;^2022^), the following elements are universally accepted: sense of being together (connection), readiness for communication (immediacy), willingness to display personal characteristics (affection). “Being together” is essential for social presence since it demonstrates that participants are present for each other in the virtual community, which signals a connection that is vital for the outcomes of the course. In the virtual environment participants tend to feel isolated and insecure so that they are reluctant to engage in class activities and more often seem to be absent. Therefore, promoting the sense of being together will be utterly challenging. Readiness for communication and willingness to display personal characteristics are closely related to each other. When participants are inclined to show their personal feelings, they feel the urge to communicate and hence know they are connected. Therefore, social presence entails social interaction, in which students make their presence known by way of socializing gestures, such as greetings, feeling-sharing, use of emojis, etc.

On the other hand, cognitive presence is composed of another dimension of interaction which involves more cognitive development and shows how students interact to make sense of the meanings or the knowledge that they try to gain from the course. Compared to teaching and social presences, cognitive presence is less researched (Sadaf, 2021) with quite a few focusing on asynchronous online learning (Garrison, 2003; Darabi, 2011; Shea & Bidjerano, 2009). According to Sadaf (2021), most researchers in recent twenty years have worked on instructional strategies that may facilitate cognitive presence, including debate, role play, scaffolding (Darabi, 2011), reflective inquiry, self-direction and metacognition(Garrison, 2003), which actually have a lot to do with teaching presence. Garrison (2000) employs four phases to analyze cognitive presence, including triggering—the experience that creates feelings of uneasiness, exploration—the knowledge-seeking process, integration—the integrating of knowledge into coherent ideas, and resolution of problems, which provide a model for teachers to help bring about adequate interaction and are widely adopted by scholars to measure cognitive presence. Meanwhile, Garrison and Cleveland-Innes (2005) emphasize that interaction is not a guarantee that students are cognitively engaged in an educationally meaningful manner. Therefore, teachers have significant roles to play in facilitating quality interaction in the community of inquiry.

### Relationship among teaching, social and cognitive presences

Understanding the complexity of interaction means to understand the interplay of social, cognitive, and teaching presences necessary for quality interaction and discourse for deep and meaningful learning (Garrison & Cleveland-Innes, 2005). The relationships among teaching, social and cognitive presences have drawn many scholars’ attention. Garrison et al. emphasize that teaching presence is crucial in creating and sustaining social and cognitive presence in online learning environments and social presence plays the mediating role between teaching and cognitive presence (2010). According to Shea and Bidjerano’s research (2009), teaching presence is a predictor of variance in learner ratings of social presence and cognitive presence and the three constructs of teaching, social, and cognitive presence are useful for describing, explaining and improving online education. Ke’s study (2010) also concludes that effective teaching presence with supportive features will reinforce the emerging of cognitive and social presence in an online learning environment. While examining the interrelationships between and among the three presences, Kozan and Richardson(2014) find that teaching presence that increases cognitive presence  will also increase social presence and cognitive presence may significantly affect the relationship between teaching presence and social presence.

Many scholars have also tried to find the role of teaching presence in various dimensions of online learning. According to Turk et al. (2022), teaching presence and social presence are also “key social-contextual support mechanisms influencing the extent to which online students of higher education perceive the satisfaction of their basic psychological needs for autonomy, competence, and relatedness.” While echoing Garrison et al.’s (2010) idea about the multiple roles of teaching presence, Szeto (2015) emphasizes its prominent role of e-teacher leadership in building up a blended synchronous community of inquiry.

In light of the aforementioned researches, teaching presence plays its unique role with the teacher’s design, facilitation and direction in promoting quality interaction. However, how the teacher fulfills such a role in large on-line literature classes demands further exploration. This study, based on the previous researches, hopes to make more contributions by focusing on how teaching presence can be created with synchronous text-based communication for adult students to achieve both social and cognitive presence, hence interact in a way that leads to satisfactory learning outcomes.

### Research design

With regard to the interaction entailed in social, cognitive presence, this study aims to explore how literature teachers may overcome the obstacle of the awkward screen and enhance teaching presence in a way to promote social and cognitive presences, and in turn facilitate quality interaction in large online class for part-time adult students.

Research questions:

1. How does teaching presence work to promote social and cognitive presence in E-learning?
2. What’s the relationship between social presence and cognitive presence? How do they account for learning outcomes and contribute to students’ actual online interaction?

To bring out the effectiveness of teaching presence, the teacher has employed some strategies to make sure teaching presence might be at work to promote social and cognitive presence. Therefore, this study aims to make sure these methods be meaningful in online literature teaching. The strategies orient towards facilitating online interaction, including student–teacher, student–student, student-content interaction. For example, before dealing with any literary text, students are required to raise questions about the text (student-content interaction) through writing synchronously on a chat platform (QQ) where everyone can see each other’s questions, then the teacher will pose her questions for them to think about. With these questions shared, students may choose to answer any student’s questions (peer interaction), or the teacher’s questions (student–teacher interaction). During this process, the teacher will give feedback to their answers in time. Eventually, the teacher will focus on some important unsolved questions and lead them to critical thinking by providing appropriate information. As the interaction is of text-based communication, it’s convenient for everyone to participate at the same time.

### Instruments and data collection

This study uses Garrison, et al.’s questionnaire (2010) and adapts it with permission to this study focusing on online literature teaching. It includes 38 items, among which 36 are questions on a 5-point Likert scale, one is a multiple-choice question about which part they enjoy most in this course, and the last one is an open question, inviting students’ feedback on the course. Besides the three parts that check teaching, social and cognitive presence respectively, six questions are related to students’ learning outcomes, such as “I realized that there was close relationship between literature and my life”, “I became interested in the three texts that we learned in class”, etc. and one is to check whether students are satisfied with this course (sense of satisfaction). This questionnaire holds a Cronbach’s Alpha reliability of 0.967. Students’ responses were  collected through the online survey platform Wenjuanxing (a questionnaire processing app). Besides, the top twenty students’ postings were  counted and analyzed to see how they actually interacted during the E-learning process.

### Participants

The questionnaires were answered by 116 part-time students of English majors who are mostly primary or middle school English teachers from all over the country and studying for Master’s Degree on education. This course of 18 class hours used to be taken in traditional face-to-face classroom during the summer. However, due to the pandemic, it’s moved to online platforms in recent years. As quite a few of the participants used to be the teacher’s students when they were undergraduates, there exists some personal presence of one another, which might be beneficial for creating higher levels of social presence and an online learning community that makes students feel connected and safe in the technology mediated learning environment.

The students were informed about the purpose of this questionnaire and were aware that the data would be used only for research and academic purposes. The participants responded in the survey anonymously. Initial results showing frequency and percentages of response in each Likert type question were automatically generated by Wenjuanxing while the other results were produced with SPSS 26.

## Results and discussion

The descriptive statistics in Table 1 show very high levels of three presences with all the means above 4. The highest mean goes to cognitive presence with students’ sense of satisfaction following closely. Social presence has the lowest mean (4.39) while cognitive presence and learning outcomes are very close to each other. The general picture delivers quite positive information about the course, since all the students have showed positive attitude (75% strongly agree, 24% agree) about whether they are satisfied with this course, except one that shows uncertainty, which is in line with students’ comments in the end of the course like “Your classes have been the most inspiring ones”, “Feel enlightened”, etc.

**Table 1:** Descriptive statistics

	N	Minimum	Maximum	Mean	SD
Teaching	116	3.89	5.00	4.7423	0.35760
Cognitive	116	3.44	5.00	4.5939	0.43912
Social	116	2.80	5.00	4.3905	0.58205
Learning outcomes	116	3.43	5.00	4.5850	0.47473
Sense of satisfaction	116	3	5	4.74	0.459

The responses also show that students have very positive attitude towards all the items in teaching presence (see Table 2). The means of Items 4 and 5 rank the highest, which shows that the teachers’ effort on increasing student involvement is well acknowledged. On the other hand, Items 6 and 10 have the lowest means, which gives another glimpse into the reality of student involvement, that’s, some students are not quite sure about their involvement. About 4.31% students show neutral attitude towards whether they are made to participate in productive dialogue, while 2.59% students are doubtful about the role of interaction with 0.89% showing disagreement.

**Table 2:** Responses to questionnaire items for teaching presence

Teaching presence	Strongly agree (%)	Agree (%)	Neutral (%)	Disagree	Strongly disagree	Mean
The instructor clearly communicated important course goals	73.28	25	1.72	0	0	4.72
The instructor was helpful in guiding us towards understanding the texts	75	24.14	0	0	0	4.74
The instructor provided clear instructions on how to participate in course learning activities	76.72	22.41	0.86	0	0	4.76
The instructor helped keep the course participants on task in a way that helped me to learn	79.31	20.69	0	0	0	4.79
The instructor encouraged us to explore the themes of the texts in this course	78.45	21.55	0	0	0	4.78
The instructor helped to keep us engaged and participating in productive dialogue	69.83	25.86	4.31	0	0	4.66
The instructor was helpful in guiding us towards understanding course topics in a way that helped me clarify my thinking	75	24.14	0.86	0	0	4.74
The instructor actions reinforced the development of a sense of community among course participants	74.14	24.14	1.72	0	0	4.72
The instructor provided feedback in a timely fashion	76.72	23.28	0	0	0	4.77
The interaction between instructor-student or among peers encouraged by the instructor stimulated me to learn	62.07	34.48	2.59	0.89	0	4.59

To understand the relationship among teaching, social and cognitive presences, learning outcomes and students’ sense of satisfaction, correlation results were  explored and indicated in Table 3.

Table 3 shows there are significant correlations among the five factors. learning outcomes are highly correlated to teaching, social and cognitive presences. There is positive correlation between teaching presence and the other four factors (all the coefficients are above 0.6), which shows students’ learning outcomes and sense of satisfaction depend to a large degree on teaching presence. The correlations between teaching presence and cognitive presence(r = 0.803, *P* < 0.01), between social and cognitive presences (r = 0.835, *P* < 0.01) are especially strong. However, what’s worth noting is that social presence seems to show relatively less correlation with students’ sense of satisfaction, which will be further explained in the later section when dealing with students’ actual interaction.

**Table 3:** Correlations between three presences and learning outcomes, sense of satisfaction

	Cognitive	Social	Teaching	Outcomes	Satisfaction
*Cognitive*					
Pearson Correlation	1	0.835**	0.803**	0.782**	0.591**
Sig. (2-tailed)		0	0	0	0
N	116	116	116	116	116
*Social*					
Pearson Correlation	0.835**	1	0.688**	0.782**	0.485**
Sig. (2-tailed)	0.000		0.000	0.000	0.000
N	116	116	116	116	116
*Teaching*					
Pearson Correlation	0.803**	0.688**	1	0.680**	0.661**
Sig. (2-tailed)	0.000	0.000		0.000	0.000
N	116	116	116	116	116
*Learning outcomes*					
Pearson Correlation	0.782**	0.782**	0.680**	1	0.618**
Sig. (2-tailed)	0.000	0.000	0.000		0.000
N	116	116	116	116	116
*Sense of satisfaction*					
Pearson Correlation	0.591**	0.485**	0.661**	0.618**	1
Sig. (2-tailed)	0.000	0.000	0.000	0.000	
N	116	116	116	116	116

**Correlation is significant at the 0.01 level (2-tailed)

As teaching presence is composed of three components—design and organization, facilitation of discourse, and direct instruction (Garrison, 2017), the correlations between the three components and social, teaching presence are analyzed to further learn how teaching presence works on social and cognitive presence(see Table 4).

**Table 4:** Correlations between three components of teaching presence and cognitive, social presence

	Design and organization (Questions 1,3)	Facilitation of discourse (Questions 4, 5, 6, 8, 10)	Direct instruction ( Questions 2, 7, 9)
*Cognitive*			
Pearson Correlation	0.674**	0.788**	0.732**
Sig. (2-tailed)	0	0	0
N	116	116	116
*Social*			
Pearson Correlation	0.541**	0.627**	0.590**
Sig. (2-tailed)	0	0	0
N	116	116	116

**Correlation is significant at the 0.01 level (2-tailed)

According to Table 4, the three components of teaching presence are significantly correlated with both cognitive and social presence. Cognitive presence is highly correlated with design and organization(r = 0.674), facilitation of discourse (r = 0.788), direct instruction(r = 0.732), while social presence is moderately correlated with design and organization(r = 0.541), direct instruction(r = 0.590). That’s to say, teaching presence has more to do with cognitive presence. Among the three components of teaching presence, facilitation of discourse has especially strong relationship with both cognitive and social presence, which shows that the teacher’ strategies in facilitating student–teacher, student–student, student-content interaction in literature classes are at work. The strong correlation between direct instruction and cognitive presence (r = 0.732) also demonstrates that the teacher’s transactional approach in her teaching has contributed to students’ cognitive engagement.

Based on the correlations, the study also hopes to find out the variance that may explain students’ learning outcomes through analyzing the regression models (see Tables 5 and 6). The three models in Table 5 show that cognitive presence alone accounts for 63% of the explained variance, which can be a very strong predictor for learning outcomes. However, teaching presence doesn’t seem to contribute much to learning outcomes with only 1.4% added variance to cognitive and social presences, which is in odds with the strong correlation between teaching presence and learning outcomes(r = 0.680). But it’s quite likely that while teaching presence has significant impact on cognitive presence (Akyol & Garrison, 2008; Kozan & Richardson, 2014), its association with learning outcomes is mediated by cognitive presence. What’s more, Table 6 shows that the residual variance for three models is far less than the explained variance, which demonstrates that the predictors (teaching, social, cognitive presence) are significant in explaining the learning outcomes.

**Table 5:** Model Summary^d^

Model	R	R square	Adjusted R square	Std. Error of the estimate	Change statistics				
					R square change	F change	df1	df2	Sig. F change
1	0.796^a^	0.634	0.630	0.28862	0.634	197.133	1	114	0
2	0.830^b^	0.690	0.684	0.26682	0.056	20.386	1	113	0
3	0.839^c^	0.703	0.695	0.26205	0.014	5.157	1	112	0.025

^a^Predictors: (Constant), cognitive

^b^Predictors: (Constant), cognitive, social

^c^Predictors: (Constant), cognitive, social, teaching

^d^Dependent Variable: learning outcomes

**Table 6:** ANOVA^a^

Model	Sum of squares	df	Mean square	F	Sig	
1	Regression	16.421	1	16.421	197.133	0.000^b^
	Residual	9.496	114	0.083		
	Total	25.917	115			
2	Regression	17.873	2	8.936	125.521	0.000^c^
	Residual	8.045	113	0.071		
	Total	25.917	115			
3	Regression	18.227	3	6.076	88.478	0.000^d^
	Residual	7.691	112	0.069		
	Total	25.917	115			

^a^Dependent Variable: learning outcomes

^b^Predictors: (Constant), cognitive

^c^Predictors: (Constant), cognitive, social

^d^Predictors: (Constant), cognitive, social, teaching

With regard to students’ actual social and cognitive presence in the form of online interaction, the postings of the students whose frequencies of interaction are in the top twenties are analyzed (see Table 7). Social presence is measured according to the three categories “personal/affective, open communication and group cohesion” proposed by Garrsion (2017). Therefore, postings that respond to the teacher’s questions about whether they can hear the teacher’s voice, whether they enjoy reading the texts or that show their gratitude to the teacher or other participants, etc. are categorized into social presence. As cognitive presence includes indicators of “sense of puzzlement”, “information exchange”, “connecting ideas”, “applying new ideas” (Garrison, 2017), raising/answering text-related questions is  counted as cognitive presence. The results show that social presence covers 42% of online synchronous interaction with a mean of 21.15 while cognitive presence covers 58% with a mean of 29.30. Obviously, more cognitive engagement is demonstrated through their cognitive interaction, which explains to a certain degree why the correlation between social presence and sense of satisfaction is rather low (0.485) compared to the other relationships. While examining cognitive presence, the frequencies of peer interaction and student–teacher interaction are also counted (see Table 8). According to the statistics, although some students show high frequencies of peer interaction (22 as the maximum), most students are not used to peer interaction with a mean of 2.1, which is consistent with students’ responses to the question about whether they enjoy answering questions raised by the other participants, as the mean of this question is 4.47, rather low compared to the other questions. This is also in line with students’ answers to the question about which part they enjoy most in this course, as 55% of the students expressed that they enjoy “the instructor’s analysis of the texts” rather than “students’ raising questions”(25%), “students’ answering questions”(12.93%) or “autonomous reading”(6.9%), which shows either the students hold more belief on the teacher(which is still a very prevalent idea among students here) or the students feel more comfortable with listening to the teacher, as peer interaction is risk taking involved in publicly espousing one’s views(Anderson, 2003). Still, students’ actual participation in raising questions is cheerful as it did stimulate more critical thinking and lead to more peer-interaction. What remains to be done might be that the teacher needs to provide more timely feedback to encourage peer interaction. Also, since they are part-time students, that’s to say, quite a few of them are busy with their work while taking this course, they cannot afford to devote more energy to their studies with listening as the easiest way, which is a pity that the teacher actually can do very little in that case. The teacher has tried to contact one student who never interacted in class (she knew she meant to be a very active participant) and learned  that she was at that time coping with a lot of work so that she had to record the teacher’s class for later studies. However, there’s no doubt that students who are paying enough attention to their peers and interact with them are likely to have stronger sense of satisfaction as they tend to feel more connected to the community and are likely to attain more cognitive development in the process. The student who has 22 times of peer interaction is a typical example, who has expressed his satisfaction and his thanks to the teacher, saying, “Thank you so much for allowing us to participate! It’s hard to feel like we’re really taking classes when facing screens too, so your efforts helped us as well!” Therefore, the teacher’s effort did pay off even though it’s quite challenging in such a big class for adult students.

**Table 7:** Descriptive statistics of online interaction of top twenty students

	N	Minimum	Maximum	Mean	SD
Social	20	4.00	35.00	21.1500	7.45001
Cognitive	20	18.00	47.00	29.3000	8.82043
Sum	20	38.00	72.00	50.4500	8.59911
Valid N (listwise)	20

**Table 8:** Descriptive statistics of peer interaction and student–teacher interaction

	N	Minimum	Maximum	Mean	SD
Peer-teacher	20	16.0	41.0	27.200	7.6406
Peer-peer	20	0	22.0	2.100	4.9407

Students’ feedback in the open question about their suggestions for this course also offered very confirmative information about online interaction, saying that they enjoyed both answering questions posted by students and learning the others’ answers and hoped that there would be more chances for students to communicate with each other.

The discrepancy between social and cognitive presence embodied in the frequencies of their actual interaction seems to contradict the strong correlation between social and cognitive presence (r = 0.832) demonstrated in the questionnaire survey, however, it confirms Garrison’ point that “social presence declined over time as cognitive and teaching presence increased” (2017), which shows the dynamics of the presences are not fixed but depends on each other’s development.

## Conclusion and recommendations

This study indicates that the teacher’s strategies for online interaction with the use of synchronous text-based communication are beneficial for promoting social and cognitive presence in large literature online classes. Teaching presence is highly correlated to social, cognitive presence as well as to learning outcomes and students’ sense of satisfaction. Social presence also has a strong relationship with cognitive presence in this E-learning environment, which is consistent with Garrison’s conclusion that “cognitive presence is more easily sustained when a significant degree of social presence has been established” (2003). Social presence and cognitive presence are strong predictors for learning outcomes which account for 68% of the explained variance in this study. Besides, students’ online interaction shows that they are more cognitively engaged rather than socially involved, which implies that students are more focused on the construction of knowledge rather than trying to be connected in the community. Also, despite the fact that students show very positive attitude towards peer interaction, there’s still a lack of it. As interaction with peers is a critical component of the formal curriculum in many disciplines (Anderson, 2003), what’s inhibiting them from interacting with each other can be a research topic for the future.

Though this study might be in need of interviews to clarify some questions presented in the survey and postings, the teacher would like to make some recommendations based on the results of the study as well as her online observation for the teachers who are teaching literature to large classes in E-learning environment. Firstly, written communication (text-based communication) is most effective to elicit responses from students as the E-space enables them to express their ideas synchronously. Since courses on literature is more involved with higher-order cognitive learning, text-based communication that allows more time for reflection is preferable to oral communication (Garrison, Anderson & Archer, 2000). Still, teachers need to learn how to provide timely feedback to encourage interaction in face of large amounts of postings. Secondly, as for part-time students who might not have fixed time for the course from time to time, asynchronous discussion can be employed to help them interact at their convenience. Thirdly, creating chances for students to tell their stories can be a viable way for them to relate to each other so that a sense of community may be fostered and social presence can be enhanced. What’s more, literature is about stories, story-telling facilitate them to interact with the texts. Lastly, splitting students into groups and keeping them on tasks may help create sociable learning environment as students are more likely to assume agency in smaller groups. In a word, online interaction is essential in distance education (Le et al., 2022), teachers have vital roles to play in promoting teaching presence and enhancing social and cognitive presences so that students may achieve satisfying outcomes through meaningful social and cognitive interaction.

## Data Availability

The raw data supporting the conclusions of this article will be made available by the author, without undue reservation.

## Abbreviations

CoI: Community of Inquiry
CMC: Computer-Mediated Communication
QQ: “Open I Seek You” chat software
et al.: And other people.
SPSS: Statistical Package for the Social Sciences
